# Omega-3 fatty acids to prevent preterm birth: Australian pregnant women’s preterm birth awareness and intentions to increase omega-3 fatty acid intake

**DOI:** 10.1186/s12937-019-0499-2

**Published:** 2019-11-14

**Authors:** Jamie V de Seymour, Lucy A Simmonds, Jacqueline Gould, Maria Makrides, Philippa Middleton

**Affiliations:** 1 SAHMRI Women and Kids, South Australian Health and Medical Research Institute, Adelaide, Australia; 2 Discipline of Paediatrics, University of Adelaide, Adelaide, Australia; 3 School of Psychology, University of Adelaide, Adelaide, Australia; 4 Robinson Research Institute, University of Adelaide, Adelaide, Australia

**Keywords:** Preterm birth, Omega-3, Nutrition, Pregnancy, Supplements, Survey translational research

## Abstract

**Background:**

Preterm birth is the leading cause of death in children under five. A recent Cochrane review found a 42% reduction in early preterm birth (< 34 weeks’ gestation) and 11% reduction in preterm birth (< 37 weeks’ gestation) with omega-3 fatty acid supplementation. To assist in the development of implementation strategies to increase pregnant women’s omega-3 fatty acid intake, we assessed the awareness of Australian pregnant women about preterm birth, their nutrition and supplementation behaviours during pregnancy, and intentions to increase omega-3 fatty acid intake.

**Methods:**

A ten-minute survey was conducted online to assess the knowledge, attitudes, behaviours, and intentions of Australian pregnant women across three domains: (1) preterm birth; (2) nutrition and supplementation during pregnancy; and (3) omega-3 fatty acid consumption to prevent preterm birth. Participants were recruited from Survey Sampling International’s research panels.

**Results:**

Of the 763 women who completed the survey, less than two-thirds had heard of preterm birth. Over 55% of respondents had changed their diet during pregnancy and a prenatal dietary supplement was consumed by 82% of the women surveyed. Respondents’ main source of information about preterm birth and nutrition during pregnancy was from a health professional. When asked about their intentions to increase their omega-3 fatty acid intake following a health professional’s recommendation, the vast majority of participants indicated they would increase their omega-3 fatty acid intake (90%). When a hypothetical scenario was presented of an omega-3 fatty acid supplement being offered from a health service at no cost, the number of respondents who selected they would increase their intake through supplementation increased from 54 to 79%.

**Conclusions:**

The main information source for women about preterm birth and dietary supplementation recommendations during pregnancy is their health professional. Therefore, informing women about ways to prevent preterm birth, including the role of omega-3 fatty acids, should occur during antenatal visits. The results from our study are useful for clinicians caring for pregnant women and for the next stage of translation of the Cochrane review findings – the design of implementation strategies to increase the intake of omega-3 fatty acids during pregnancy where needed.

## Introduction

Preterm birth is a pregnancy complication that can result in short- and long-term consequences, including problems with respiratory, gastrointestinal, and immune system function; vision and hearing; and cognitive and behavioural problems [1, 2]. Preterm birth is the global leading cause of death in children under five [3].

A number of studies have investigated omega-3 fatty acids for their potential to prevent preterm birth, stemming from findings that, compared to their Danish neighbours, Faroe Islanders had longer gestations and heavier babies, and a significantly higher fish intake [4]. A Cochrane review synthesising evidence from 70 randomised controlled trials of omega-3 fatty acid supplementation during pregnancy showed that omega-3 fatty acid intervention during pregnancy reduced the risk of preterm birth by 11% and early preterm birth (birth < 34 weeks’ gestation) by 42% [5]. These findings have the potential to be translated into a simple and effective population-based preterm birth reduction strategy with global health impact.

The key omega-3 fatty acids in the Cochrane review were the marine-based long-chain polyunsaturated fatty acids, docosahexaenoic acid (DHA) and eicosapentaenoic acid (EPA). The main natural dietary source of DHA and EPA are oily fish, such as salmon, mackerel, sardines, and trout. To obtain the dose of DHA that was highlighted as effective for reducing preterm birth in the review, a pregnant woman would need to eat at least two serves (~ 150 g per serve) of salmon each week. A study of fish consumption in Australian women found that intake during pregnancy was, on average, less than 200 g of total fish (oily and non-oily) per week [6]. Pregnant Australian women may need to change their diets to obtain the dose recommended in the Cochrane review through consumption of omega-3 fatty acid rich foods, such as fish. Omega-3 fatty acid supplements offer a convenient and simple alternative to dietary change, and nutritional supplements are generally well accepted by Australian pregnant women [7, 8].

Given the potential to reduce preterm birth highlighted in the recent Cochrane review, we assessed the current awareness of Australian pregnant women about preterm birth, and their nutrition and supplementation behaviours during pregnancy. Additionally, we presented women with information about the possible prevention of preterm birth through omega-3 fatty acid supplementation, and then asked their intentions to increase omega-3 fatty acid intake during pregnancy.

## Methods

### Research design and measures

A cross-sectional research design was performed among Australian pregnant women (or those who had given birth within the previous six months). A 43-item survey was developed based on best practice principles [9] and evaluated by health professionals and women who had given birth to assess the knowledge, attitudes, behaviours, and intentions of pregnant women (survey items listed in the Additional file 1). The survey was subsequently pilot tested by 100 participants for feasibility. Two screening questions were first included to exclude women who indicated they were not pregnant or had not recently given birth, or who did not agree to participate after reading the participant information sheet. In addition to demographic data (15 items), information was collected across three domains: (1) preterm birth; (2) nutrition and supplementation during pregnancy; and (3) omega-3 fatty acid consumption to prevent preterm birth. In the final stage of the survey, respondents were asked about behavioural intentions to change their omega-3 fatty acid intake following presentation of information on preterm birth and its consequences, an omega-3 fatty acid evidence statement and recommendation. Throughout the survey, omega-3 fatty acids were referred to as omega-3 s. Responses to the key survey items are detailed in the results section.

Ethical approval for this study was granted by the Women’s and Children’s Health Network Human Research Ethics Committee in Adelaide, Australia (HREC/17/WCHN/155). An information sheet outlining the purpose of the survey was provided with the link to the survey. Completing the online survey implied consent.

### Study respondents

Respondents were recruited through Survey Sampling International’s (now Dynata) research panels and responses to the online survey were collected between August 2018 and January 2019. Women were eligible for the survey if they were currently pregnant or had given birth within the last six months, were over the age of 18 years, living in Australia, and willing to participate in the study. Women were excluded if they indicated at the screening question that they were currently pregnant but answered “0” when asked their gestational age.

### Statistical analysis

Data coding and analyses were performed using SPSS (SPSS, version 25.0, Armonk, NY, USA). Descriptive statistics were reported as frequencies and percentages; or medians and lower and upper quartiles. The distributions of continuous variables were assessed for normality using the Shapiro-Wilk test. As data were not normally distributed, non-parametric tests were conducted when testing for differences between groups; the Mann Whitney *U* test was used for continuous variables and Fisher’s exact test (chi-square) was used for categorical variables. All analyses were two-sided and statistical significance was set at a *P*-value less than 0.05. Subgroup analyses were performed on relevant questions to investigate differences in responses between nulliparous and multiparous women.

## Results

### Respondent characteristics

Seven hundred and sixty three women (*n* = 763) completed the survey. Demographic characteristics are displayed in Table 1. The median (interquartile range) age and body mass index (BMI) of respondents was 30 years (26–34) and 24.1 kg/m^2^ (20.8–29.0), respectively. Overall, 87.9% of respondents indicated that they had completed high school; 84.8% of those completing high school had completed further education. Women expecting their first baby were significantly younger than those who had previously given birth, and were more likely to have completed high school.

**Table 1:** Demographic characteristics

Characteristics	All Respondents (*N* = 763)	Nulliparous Respondents (*N* = 308)	Multiparous Respondents (*N* = 455)	Australian Pregnant Women^3^
Age (years)^1^ *	30 (26-34)	29 (25-33)	31 (27-34)	30.5^#^
Body mass index (kg/m^2^)^1^	24.1 (20.8-29.0)	23.7 (20.5-28.3)	24.3 (21.1-29.3)	25.7^#^
Aboriginal and/or Torres Strait Islander^2^	58 (7.6)	29 (9.4)	29 (6.5)	4.4%
Born in Australia^2^	613 (80.3)	238 (77.3)	370 (82.4)	65.1%
Number of children in household^1*^	1 (0-2)	0 (0-0)	1 (1-2)	
Total in household^1*^	3 (2-4)	2 (2-3)	3 (3-4)	
Weeks of gestation^1^	21 (12-30)	22 (12-32)	21 (12-29)	
Completed high school^2*^	671 (87.9)	286 (92.9)	379 (84.4)	89.0%^4^
Completed further education^2^	601 (84.8)	252 (86.3)	344 (83.7)	
Further education^2^:				
Trade certificate	90 (15.0)	44 (17.5)	46 (13.4)	
Diploma or certificate	214 (35.6)	80 (31.7)	132 (38.4)	
Degree	235 (39.1)	105 (41.7)	127 (36.9)	
Higher degree	112 (18.6)	49 (19.4)	62 (18.0)	
Other	9 (1.5)	4 (1.6)	5 (1.5)	
Planned pregnancy^2^	535 (70.1)	227 (73.7)	303 (67.5)	
Smoked during pregnancy^2^	107 (14.0)	38 (12.3)	68 (15.1)	9.7%
Expecting first child^2^	308 (40.4)	308 (40.4)	N/A	42.8%
State^2^				
Australian Capital Territory	15 (2.0)	5 (1.6)	10 (2.2)	2.1%
New South Wales	244 (32.0)	87 (28.2)	156 (34.7)	31.4%
Northern Territory	6 (0.8)	4 (1.3)	2 (0.4)	1.3%
Queensland	170 (22.3)	67 (21.8)	100 (22.3)	19.9%
South Australia	59 (7.7)	22 (7.1)	36 (8.0)	6.4%
Tasmania	23 (3.0)	11 (3.6)	12 (2.7)	1.9%
Victoria	190 (24.9)	89 (28.9)	100 (22.3)	25.7%
Western Australia	56 (7.3)	23 (7.5)	33 (7.3)	11.4%

^1^ Median (interquartile range)

^2^Number of respondents (N) (% of total respondents)

^3^Data obtained from *Australia’s mothers and babies 2016* report [10]

^4^Number of female students staying in school until Year 12. Data obtained from Report 4221.0, The Australian Bureau of Statistics [11]

Mean Chi-square tests were performed to test for differences between categorical variables and Mann-Whitney U tests were performed for continuous variables, when comparing nulliparous and multiparous groups

* *P* < 0.05

Although the respondents in this study had similar demographic characteristics to Australian women delivering in 2016 [10], the women included in this study were more likely to be born in Australia, identify as Aboriginal and/or Torres Strait Islander, and were more likely to indicate they had smoked during pregnancy.

### Preterm birth knowledge

Only 61.6% of respondents had heard of preterm birth, whereas 84.0% had heard of premature birth. Multiparous women were significantly more likely to have heard of preterm birth than nulliparous women (65.7% vs 55.5%; *P* = 0.02). Overall, 52.7% indicated they were most familiar with the term “premature birth” out of the four synonyms presented (“preterm birth”, “premature birth”, “premmie baby”, and “prem baby”). Only 37.9% of the respondents correctly defined premature birth as “birth before 37 weeks of gestation”, when provided with eight response options (ranging from “birth before 32 weeks” to “birth before 42 weeks”, and the option of “a baby born with a low birthweight”). Multiparous women were significantly more likely to correctly answer the definition of premature birth than nulliparous women (43.0% vs 30.5%; *P* = 0.02).

Of the women who had heard of preterm birth (or its synonyms) before commencing the survey, the main source of information about preterm birth was through a health professional (*n* = 290; 55.8%). The next most common source was the internet (*n* = 179; 34.4%), followed by family (*n* = 153; 29.4%) and friends (*n* = 135; 26.0%).

### Maternal dietary practices and supplement use

Of the 763 women surveyed, 432 (56.6%) expressed that they had changed their diet for this pregnancy. Nulliparous women were significantly more likely to indicate they had changed their diet (66.9% vs 49.4%; *P* < 0.001).

A dietary supplement was consumed during their current pregnancy by 625 (81.9%) of the women surveyed and nulliparous women were more likely to be consuming a dietary supplement than multiparous (87.0% vs 78.4%; *P* = 0.01). The nutrient supplement most commonly consumed by respondents was a pregnancy multivitamin (73.4%), followed by folic acid (48.3%). The main motivation for deciding to consume a nutrient supplement during pregnancy was “for the health of the baby” (71.7%), closely followed by the influence of an external recommendation (68.8%), and “for maternal health and well-being” during pregnancy (60.0%) (Table 2).

**Table 2:** Maternal motivations for choosing to take a nutrition supplement/s during pregnancy. (Respondents were asked to select all options that applied to them)

Motivation	*N* (%)
For the health of my baby	448 (71.7)
Advice given to me	430 (68.8)
To keep me healthy during pregnancy	375 (60.0)
A supplement was the easiest way to get the nutrients I need	163 (26.1)
I took supplements in my other pregnancies^a^	136 (35.8)
I’ve seen/heard that other pregnant women are taking it	109 (17.4)
The supplements were given to me	22 (3.5)
Other	7 (1.1)

^a^Calculated as the % of multiparous women who indicated they took a nutrition supplement during pregnancy

**Fig. 1:**
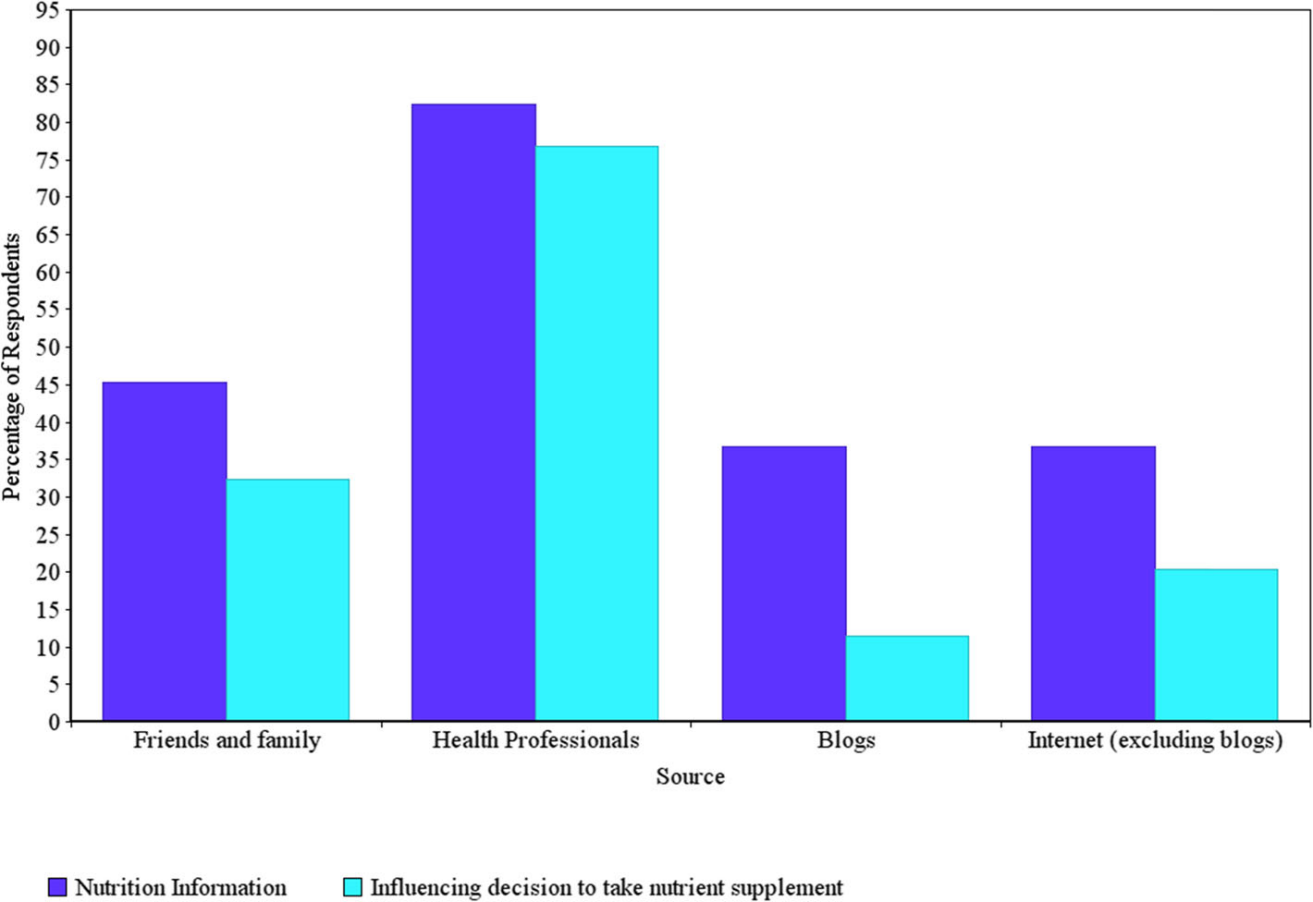
Comparing key sources used to obtain nutrition information during pregnancy with key sources that influenced maternal decision-making to consume a nutrient supplement (or not) during pregnancy

Respondents sought information on nutrition during pregnancy from a range of sources: the most common sources of information were health professionals (82.3%) and information from friends and family members (45.3%). When asked about who has the greatest influence on their decision to take or not take nutrient supplements during pregnancy, respondents nominated health professionals, and friends and family members (76.8 and 32.4%, respectively). Although pregnancy blogs and the general internet were selected by respondents as sources of nutrition information, they were much less likely to indicate these sources as key influencers in their decision to take supplements in pregnancy (Fig. 1).

### Omega-3 fatty acids

A high proportion of respondents (90.6%) had heard of omega-3. Omega-3 fatty acid containing supplements had been consumed by 37.7% of respondents in the 12 months prior to conceiving and 30.4% indicated that they had consumed an omega-3 fatty acid containing supplement during their current pregnancy. Less than a third of the women surveyed (32.1%) were eating at least two serves of fish a week and 19.3% indicated they never ate lean or fatty fish during their pregnancy.

When asked about their intentions to increase their omega-3 fatty acid intake following a government guideline and health professional’s recommendation, a high proportion indicated they would increase their omega-3 fatty acid intake (89.5%), with 53.7% preferring to take an omega-3 fatty acid supplement and 35.8% preferring to increase their omega-3 fatty acid intake from food sources (Table 3). Of the 10.5% (*n* = 80) who indicated they would not make any changes to their omega-3 intake, about a third felt they already had a good diet/were getting enough omega-3 fatty acids (*n* = 26; 32.5%,). However, only two (7.7%) of the respondents who felt they were already getting enough omega-3 fatty acids ate two or more serves of fatty fish per week.

**Table 3:** Maternal intentions following introduction of key facts about preterm birth and the evidence of omega-3 fatty acids to reduce the risk of preterm birth

Intention	*N* (%)
Choose to take an omega-3 fatty acid supplement during pregnancy	410 (53.7)
Choose to change diet to increase omega-3 fatty acids from foods during pregnancy	273 (35.8)
Not make any changes to diet or supplement intake	80 (10.5)

Although roughly half (53.7%) of respondents indicated they would increase their omega-3 fatty acids by taking an omega-3 fatty acid supplement during pregnancy, if an omega-3 fatty acid supplement were offered at no cost from a health service, 78.8% of respondents indicated they were likely or highly likely to do so (Table 4).

**Table 4:** Respondents’ intention to take an omega-3 fatty acid supplement at no cost during pregnancy^a^

Intention	*N* (%)
5	370 (48.5)
4	231 (30.3)
3	109 (14.3)
2	29 (3.8)
1	24 (3.1)

^a^Respondents’ rating out of 5 (1 = highly unlikely to 5 = highly likely) when asked how likely they would be to take an omega-3 fatty acid supplement during pregnancy if it were offered for free from health professionals

## Discussion

In this study, we assessed the current knowledge of Australian pregnant women regarding preterm birth, their nutrition and supplementation behaviours during pregnancy, and their intended behaviours to supplement with omega-3 fatty acids to reduce the risk of preterm birth. A key finding was the low level of awareness of preterm birth, its definition, and its consequences. This was an unexpected finding in a cohort of well-educated pregnant women. This suggests that preterm birth awareness needs to be increased in Australia.

Once presented with information on the consequences of preterm birth and the evidence – that the risk of preterm birth may be reduced by increasing omega-3 fatty acid intake – a high proportion of respondents indicated they were willing to increase their omega-3 fatty acid consumption during pregnancy. While about a third of women said they would prefer to increase their omega-3 fatty acid intake through food sources, less than a fifth of the women were eating the amount of fatty fish required to meet the recommended omega-3 fatty acid intake to prevent preterm birth. These low levels of fish intake in Australian pregnant women are in line with the findings of Taylor, Collins, & Patterson [6]. The discordance between respondents’ current behaviours and their intended behaviours to meet the dose of omega-3 fatty acids to reduce the risk of preterm birth highlighted in the Cochrane review suggests that dietary education in pregnancy around omega-3 fatty acids should accompany information provided on preterm birth.

Over half of the respondents reported they had already changed their diet during pregnancy and a substantial majority indicated they had consumed a dietary supplement during their current pregnancy. Similarly high rates of supplement use during pregnancy have been reported in a previous study conducted in Melbourne, Australia (91%) [7]. Nulliparous women were significantly more likely to have changed their diet during their pregnancy and were more likely to be taking a dietary supplement to support their nutritional needs than multiparous women. This finding may be partially explained by the differences between the two groups in reasons for supplementing during pregnancy. Nulliparous women were significantly more likely to cite their main reason for supplementing was due to advice given to them or because they had seen/heard of other pregnant women taking them.

This survey captured novel information which will contribute towards the design and implementation of strategies to increase the intake of omega-3 fatty acids in pregnant women. Health professionals were identified by respondents as their main source for information on preterm birth, nutrition during pregnancy, and influencing their decision to take a nutrient supplement. This strong link with health professionals in antenatal care as a trusted source of information and guidance highlights antenatal visits as a promising avenue to deliver information on preterm birth and evidence-based strategies to reduce the risk of preterm birth. An effective implementation strategy might include updating the clinical practice guidelines and educating health professionals in antenatal care to encourage an increase in omega-3 fatty acid consumption during pregnancy.

A key strength of this research was the large number of Australian women included in the study. As with any self-reported data, there are some risks of bias influencing the women’s responses to some of the questions in the survey. In particular, social desirability bias and recall bias might have affected the survey results, though this is difficult to assess.

## Conclusion

The results from our study have uncovered important factors that could be addressed to increase the intake of omega-3 fatty acids by pregnant women. These findings can be used to inform the design and implementation of any strategies seeking to increase the intake of omega-3 fatty acids during pregnancy in order to reduce preterm birth. We found that the main information source for women about preterm birth and dietary supplementation recommendations during pregnancy is their health professional, emphasising the need to inform women about ways to prevent preterm birth, including the possible role of omega-3 fatty acids, during antenatal visits.

## Supplementary information

**Additional file 1.** Survey items included in the online survey.

## Data Availability

The datasets used and/or analysed during the current study are available from the corresponding author on reasonable request.

## Abbreviations

BMI: Body mass index
DHA: Docosahexaenoic acid
EPA: Eicosapentaenoic acid
