# Supplementary material for: Omega-3 fatty acids to prevent preterm birth: Australian pregnant women’s preterm birth awareness and intentions to increase omega-3 fatty acid intake
Source: Nutr J. 2019 Nov 14;18:74. doi: 10.1186/s12937-019-0499-2 (PMC6857157; doi:10.1186/s12937-019-0499-2)
Supplement: Supplementary file 1 — Additional file 1. Survey items included in the online survey. [file 12937_2019_499_MOESM1_ESM.docx]

**Survey items included in the online survey**

**Screening questions**

Please confirm: I have read and understood the information sheet and agree to participate in this research?

Yes *-> proceed to second screening question*

No *-> excluded from survey*

Are you currently pregnant or have given birth within the previous 6 months?

Yes, currently pregnant *-> proceed to survey*

Yes, gave birth within the previous 6 months *-> proceed to survey*

No *-> excluded from survey*

**Pregnancy-related terminology**

1. Have you heard of any of the following terms? (please select all that apply).

preterm birth

premature birth

premmie baby

prem baby

none of the above

2. Which term are you most familiar with?

preterm birth

premature birth

premmie baby

prem baby

I don't know any of these

Other (please specify)

3. How long should a pregnancy normally last?

36 weeks

37 weeks

38 weeks

39 weeks

40weeks

41 weeks

42 weeks

I don't know

Other (please specify)

4. What is the definition of preterm birth? (please select the answer that best fits).

birth before 32 weeks of pregnancy

birth before 34 weeks of pregnancy

birth before 37 weeks of pregnancy

birth before 39 weeks of pregnancy

birth before 40 weeks of pregnancy

birth before 41 weeks of pregnancy

birth before 42 weeks of pregnancy

A baby with a low birthweight

I don't know

Other (please specify)

5. What is the definition of premature birth? (please select the answer that best fits).

birth before 32 weeks of pregnancy

birth before 34 weeks of pregnancy

birth before 37 weeks of pregnancy

birth before 39 weeks of pregnancy

birth before 40 weeks of pregnancy

birth before 41 weeks of pregnancy

birth before 42 weeks of pregnancy

A baby with a low birthweight

I don't know

Other (please specify)

6. When is a "premmie or prem baby" born? (please select the answer that best fits).

birth before 32 weeks of pregnancy

birth before 34 weeks of pregnancy

birth before 37 weeks of pregnancy

birth before 39 weeks of pregnancy

birth before 40 weeks of pregnancy

birth before 41 weeks of pregnancy

birth before 42 weeks of pregnancy

I don't know

Other (please specify)

**Omega-3 fatty acids**

7. Have you heard of omega-3s?

yes

no

8. Have you heard of omega-3 fats?

yes

no

9. Do you know what food sources contain omega-3 fats?

yes

no

I know some sources

I don't know

9a. If yes or I don’t know, which of the following foods do you think contain high amounts of omega-3 fats? (please select all that apply).

chicken and poultry

fatty fish (e.g. salmon, mullet, bream, trout, sardines, herring, mackerel)

lean fish (e.g. basa, snapper, whiting, ling, barramundi)

red meat (e.g. beef and lamb)

seafood (not including fish)

fish oils

algae/seaweed

green leafy vegetables

red and orange coloured vegetables

fruit

bread

butter

eggs

milk and other dairy products

nuts and seeds

I don't know

Other (please specify)

**Demographic questions**

10. Which state/territory do you currently live in?

Australian Capital Territory

New South Wales

Northern Territory

Queensland

South Australia

Tasmania

Victoria

Western Australia

11. What year were you born?

12. What is your usual pre-pregnancy weight (approximately)? Please answer in kilograms.

13. What is your height (centimetres)?

14. Did you complete high school?

yes

no

15. Have you completed any further studies since you left high school?

yes

no

Other (please specify)

16. What further studies have you completed? (please select all that apply)

completed a trade certificate/apprenticeship

completed a diploma or other certificate

completed a degree

completed a higher degree (e.g. post-graduate diploma, masters, PhD)

Other (please specify)

17. Do you identify as an Aboriginal or Torres Strait Islander person?

yes

no

18. Were you born in Australia?

yes

no

If no, in which country were you born?

19. How many children (under 18 years) do you have living at home with you?

20. How many people are living in your household (including you)?

21. Approximately how many weeks pregnant are you?

22. Was this pregnancy planned?

yes

no

23. Are you expecting your first child?

yes

no

Other (please specify)

24. Have you smoked at all during pregnancy?

yes

no

**Nutrition and supplementation during pregnancy**

25. Have you changed your diet for this pregnancy?

yes

no

26. Where do you get your information about nutrition during pregnancy? (please select all that apply).

friends

family

health professionals (GP, midwife, obstetrician, health worker)

dietitian/nutritionist

pregnancy/ parenthood/ motherhood blog(s)

social media

internet (excluding blogs)

TV

pregnancy books magazines or newspapers

I haven't sought any information about nutrition during pregnancy

I don't remember

Other (please specify)

26a. If answered yes to 'blog/s', please name which blog(s) or blogger(s) you read or follow

27. Did you take any nutrient supplements in the 12 months before you were pregnant?

yes

no

28. Have you taken any nutrient supplements during your pregnancy?

yes

no

28a. If yes, please select from the list below which nutrient supplements you have taken during your pregnancy (please select all that apply).

folic acid

iodine

vitamin D

iron

a pregnancy multivitamin (eg. Elevit, Blackmore's Pregnancy and Breastfeeding Gold, Nature's Own Pregnancy Platinum, Swisse Pregnancy Ultivite Multivitamin)

Other (please specify)

28b. If yes, what are the main reason/s you decided to take nutrient supplements during your current pregnancy? (please select all that apply).

advice given to me

I've seen/heard that other pregnant women are taking it

for the health of my baby

to keep me healthy during pregnancy

a supplement was the easiest way to get the nutrients I need

I took supplements in my other pregnancies

the supplements were given to me

Other (please specify)

29. What influenced your decision to take or not take nutrient supplements during pregnancy? (please select all that apply).

advice from family, friends, or colleagues

advice from health professional (GP, midwife, obstetrician, health worker)

information from the internet

advertising on TV, in-store, or on supplement packaging

information in pregnancy books, magazines or newspapers

information on pregnancy / parenthood / motherhood blogs social media

Other (please specify)

30. Do you have any concerns about taking supplements in pregnancy? If so, please state.

yes

no

If yes, please detail your concerns about taking supplements in pregnancy.

31. Have you consumed omega-3 supplements (fish oil/calamari oil/algal oil/krill oil) before you were pregnant?

yes

no

32. Have you consumed any omega-3 supplements (fish oil/calamari oil/algal oil/krill oil) during your pregnancy?

yes

no

33. Are you continuing to take omega-3 supplements during your pregnancy?

yes

no

33a. If you have previously taken omega-3 supplements but have now stopped, what was your reason/s for stopping? (please select all that apply).

Cost

fishy burps

taste

nausea or morning sickness

worried that the supplements may have been rancid ("gone off") or oxidized

I already get enough from my food

advice I received from a friend, family member, or colleague to stop taking them

ethical/sustainability reasons (e.g. worried about impact on the global fish stocks)

advice I received from a health professional (e.g. doctor, midwife, obstetrician) to stop taking them

I kept forgetting to take them

I don't like swallowing pills

too much hassle

worried about the supplements containing contaminants (e.g. heavy metals such as mercury)

Other (please specify)

34. During this pregnancy, how many times, on average, do you eat the following foods?

34a. Lean fish or seafood (e.g. oysters, mussels, prawns, basa, canned tuna, snapper, whiting, ling, barramundi).

more than once a day

6-7 times a week

4-5 times a week

2-3 times a week

once a week

1-3 times a month

less than once a month

never

34b. Fatty fish (e.g. salmon, mullet, bream, trout, sardines, herring, mackerel).

more than once a day

6-7 times a week

4-5 times a week

2-3 times a week

once a week

1-3 times a month

less than once a month

never

34c. Lean red meat (e.g. kangaroo, venison, beef or lamb with visible fat removed).

more than once a day

6-7 times a week

4-5 times a week

2-3 times a week

once a week

1-3 times a month

less than once a month

never

35. Do you eat omega-3 enriched eggs?

yes

no

don't know

36. Do you eat any other omega-3 enriched products (e.g. Uncle Tobys Plus Omega-3 Cereal, The One Tip Top Omega-3 Sandwich Bread, Tasmania's Westhaven Omega-3 Yoghurt, OmegaMite Yeast Spread)?

yes

no

don't know

**Preterm birth**

37. Do you know the possible negative effects of preterm birth on the child's health?

yes

no

I think I know some

I think I know one

not sure

37a. If yes or I think I know some, what is a possible negative effect of preterm birth on a child's health?

37b. If any but no, where did you get information on preterm birth? (please select all that apply).

friends

family

health professional (GP, midwife, obstetrician, health worker)

pregnancy/ parenthood/ motherhood blog(s)

social media

internet (excluding blogs)

TV

pregnancy books magazines or newspapers

I don't remember

Other (please specify)

38. Has your health professional (GP, midwife, obstetrician, health worker) spoken to you about preterm birth during this pregnancy?

yes

no

38a. If yes, approximately how many weeks pregnant were you when your health professional spoke to you about preterm birth?

39. Have you, or someone that you know well, previously had a preterm birth (before 37 weeks of pregnancy)?

yes

no

**Behavioural intentions**

Preterm birth is when a baby is born before 37 weeks of pregnancy.

Preterm birth may have negative effects on the child's health in the short- and long-term, including:

• Problems with their lungs, gut, and immune system function

• Problems with their vision and hearing

• Problems with behaviour, learning, and communicating with others

There are also emotional and economic costs to families of babies born too soon.

“Evidence from a large number of research studies has shown that omega-3 supplementation during pregnancy lowers the risk of having a premature baby, mostly in women carrying a single baby. "

40. If the statement above was from government guidelines, and health professionals were recommending that you increase your omega-3 intake in pregnancy, would you:

choose to take an omega-3 supplement during pregnancy

choose to change your diet to increase your omega-3 fats from foods during pregnancy

not make any changes to your diet or supplementation intake

40a. If you would choose to take an omega-3 supplement, what is the reason/s for your answer? (please select all that apply).

cost of foods high in omega-3

access to foods high in omega-3

I don’t know what foods are high in omega-3

I don't like the foods that are high in omega-3

I have concerns about mercury risks or other contaminants in fish or seafood high in omega-3

I'm vegetarian so would prefer to get my omega-3 from algal oils

struggling with nausea or sickness so can't tolerate foods high in omega-3 such as fish and seafood

Other (please specify)

40b. If you would choose to change your diet, what is the reason/s for your answer? (please select all that apply).

cost of omega-3 supplements

access to omega-3 supplements

I'm struggling with nausea or sickness so swallowing pills can be difficult

I don't know which omega-3 supplement I should choose

I have concerns about mercury risks or other contaminants in omega-3 supplements

I have concerns about the environment (e.g. worried about impact on global fish stocks)

I'm vegetarian and most of the omega-3 supplements are from fish oils

I can’t swallow capsules

it is better to get nutrients in food form

Other (please specify)

40c. If you would choose to not make any change, what is the reason/s for your answer? (please select all that apply).

cost of increasing omega-3 through supplements or food

access to omega-3 supplements or foods high in omega-3

I'm struggling with nausea or sickness

I have concerns about mercury risks or other contaminants in omega-3 supplements and food sources

I have concerns about the environment (e.g. worried about impact on global fish stocks)

I would want to do my own research before deciding to increase my omega-3

too much effort

I already have a good diet/get enough omega-3

Other (please specify)

41. If there was an omega-3 supplement that was endorsed as having the right amounts of omega-3 and was safe to take during pregnancy, who's endorsement would influence you to take an omega-3 supplement during pregnancy? (please select all that apply).

a government agency (e.g. National Health and Medical Research Council)

research institute (e.g. South Australian Health and Medical Research Institute)

a pregnancy organisation (e.g. Pregnancy, Birth and Baby Australia)

college of midwives

college of obstetricians

a consumer group (e.g. miracle babies)

none of the above

Other (please specify)

42. If omega-3 supplements (from fish oil OR algae-based if vegetarian) were provided free of charge at your first antenatal visit, how likely would you be to take them throughout pregnancy? Please select between 1 (not at all likely) to 5 (very likely).

43. What is the reason/s for your answer in the last question? (please select all that apply).

they're free

it has been recommended to me

I’d like to research them before taking them

I’d listen to the recommendation but choose my own brand

I don't take nutrient supplements

I would rather get the recommended omega-3s through food

it would depend on who the supplement manufacturer/supplier was

I don't think it will help me/I don't think it will work

Other (please specify)

Thank you for completing the survey. If you have any feedback about the survey, please leave your comments in the box below.
